# Developmental Regulation of Corazonin, Eclosion Hormone, and Bursicon Messages and RNAi Suppression of Corazonin in Adult, Female American Dog Ticks, *Dermacentor variabilis*

**DOI:** 10.3390/insects16040343

**Published:** 2025-03-25

**Authors:** Anirudh Dhammi, Brooke Bissinger, Loganathan Ponnusamy, Daniel E. Sonenshine, R. Michael Roe

**Affiliations:** 1 Department of Entomology and Plant Pathology, North Carolina State University, Raleigh, NC 27695, USA; anirudhdhammi@gmail.com (A.D.); brooke.bissinger@basf.com (B.B.); lponnus@ncsu.edu (L.P.); 2 Department of Biological Sciences, Old Dominion University, Norfolk, VA 23529, USA; daniel.sonenshine@nih.gov

**Keywords:** ticks, *Dermacentor variabilis*, neuropeptides, corazonin, eclosion hormone, α bursicon, β bursicon, qPCR, RNAi

## Abstract

The insect molting process is critical to growth and development and is regulated in part by the neuropeptides corazonin, eclosion hormone, and α and β bursicon. We found messages in a synganglion transcriptome from adult, female American dog ticks, *Dermacentor variabilis* (that do not molt), with a high similarity to the larval insect neuropeptides that control molting. The phylogenetic analysis of the tick putative neuropeptides compared to other arthropods is discussed in detail. The relative gene expression of these peptides was determined by quantitative PCR during the following adult developmental stages: (i) virgin, unfed 0–24 h after entering the adult stage (non-host-seeking), (ii) host-seeking, unfed, and not mated (3 d after emergence), (iii) part-fed (unmated, attached to host; 1st and 3rd day after emergence), (iv) mated (females are part-fed; allowed to mate for ≤1 day, 7th day after emergence), (v) mated repletes (completion of blood feeding but still attached to host), and (vi) post-drop-off (from host) with egg laying starting within 1 d of detachment. Eclosion hormone transcript levels peaked at mating and at drop-off. Bursicon α levels were highest just after molting into adults, with a second smaller peak in replete females. Bursicon β levels were highest (32-fold) post-drop-off. Corazonin message levels peaked in part-feds and were much higher (40-fold) in repletes compared to 0–24 h after emergence. RNAi suppression of the corazonin message by injection in newly molted ticks reduced oviposition and the number of vitellogenic eggs in the ovaries at drop-off but had no apparent effect on host-seeking, partial feeding, mating, feeding to repletion, and drop-off. The possible roles of these transcripts in adult, female tick development are discussed.

## Simple Summary

The insect molting process including their shedding of the old cuticle and hardening of the new cuticle is regulated by a cascade of peptide hormones, including corazonin, eclosion hormone and α and β bursicon. The messenger RNA for these hormones were found in adult, female American dog ticks. Since adult ticks do not molt, this finding suggests the hormones that regulate insect molting might also control female reproduction. Changes in the developmental expression levels of the messages of these hormones during tick host seeking, blood feeding, mating and oviposition argues they are involved in reproduction. The artificial suppression of corazonin that initiates the molting cascade reduced the maturation level of eggs and reduced egg deposition in the American dog tick.

## 1. Introduction

Ticks are ectoparasites and vectors of many pathogens that cause diseases, including Lyme disease and Rocky Mountain spotted fever in humans [1]. In addition, ticks are an economically important ectoparasite of livestock and companion animals. Despite the progress in genetics and livestock management, tick control relies heavily on acaricides that target the nervous system [2,3]. Unfortunately, like in insects, ticks are developing resistance to pesticides used to control them [3,4], and this is why we need to identify new targets for tick control.

Little is known about the endocrinology of ticks compared to insects. Assumptions about the regulation of growth, molting, metamorphosis, and reproduction in arthropods is based mostly on our knowledge on insects, and in some cases, these assumptions are not supported. For example, metamorphosis and reproduction in ticks for many years were assumed to be the same as in insects, controlled by juvenile hormone (JH) [5,6]. However, Neese et al. [7] found that ticks did not synthesize JH, and no JH was found in both soft and hard ticks by *Galleria* bioassay and GC-EI mass spectrometry. Fundamental research is needed in tick endocrinology, which is many decades behind research on insects and could lead to new technology for tick control.

The molting pathway has been well studied in insects. Pre-ecdysis, ecdysis, and post-ecdysis (molting) behavior and cuticle sclerotization after ecdysis are regulated by peptide hormones, i.e., corazonin, pre-ecdysis-triggering hormone (PETH), ecdysis-triggering hormone (ETH), crustacean cardioactive peptide (CCAP), eclosion hormone (EH), and bursicon α and β [8]. Declining ecdysteroid levels induce expression of the corazonin receptor in Inka cells and allow the release of the neuropeptide corazonin from the brain–corpora cardiaca–corpora allata complex. A peptide-driven endocrinological cascade results in ecdysis (shedding of the old cuticle) and hardening of the new cuticle. The appearance and specific interactions that occur between these peptides at specific times and amounts are critical to successful molting [8,9]. Corazonin is an undecapeptide hormone, which was originally discovered as a cardioaccelerator in the cockroach *Periplaneta americana* [10]. Even though it functions in cockroaches as a cardioaccelerator, it has been found in other insects with different functions [11,12,13]; its role in ecdysis is well understood [8,14]. Eclosion hormone (EH) from the insect brain stimulates the release of PETH and ecdysis-triggering hormone (ETH). Eclosion hormone (EH) is a neuropeptide discovered in *Manduca sexta* [15] and produced by pairs of neurosecretory cells in the ventromedial protocerebrum [16]. ETH released from Inka cells activates the entire network of peptidergic neurons that controls the ecdysis sequence. Kinins and diuretic hormones initiate pre-ecdysis, while the central release of eclosion hormone, CCAP, bursicon, and other peptides induces ecdysis and post-ecdysis behaviors. ETH released from Inka cells triggers the critical process of shedding of the exocuticle [17,18]. After ecdysis, tanning and sclerotization are controlled by α and β bursicon [8]. Bursicon is a heterodimer that consists of two cysteine knot proteins, i.e., α and β bursicon, which are released from the thoracic ganglia [19,20,21].

Interestingly, we found transcripts similar to the insect peptide molting hormone messages, i.e., corazonin, eclosion hormone, and α and β bursicon, in a synganglion transcriptome of adult, female American dog ticks, *Dermacentor variabilis* [22]. This was unexpected when first discovered since adult ticks do not molt. Since our discovery of these messages in adult *D. variabilis*, they have been reported elsewhere in other adult ticks. For example, Waldman et al. [23] found transcripts for the insect molting peptide hormones, i.e., corazonin, eclosion hormone, ecdysis-triggering hormone (ETH), crustacean cardioactive peptide (CCAP), and α and β bursicon, in the synganglion transcriptome of adult, female *Rhipicephalus microplus* ticks [23]. Messages for a partial neuropeptide molting pathway, i.e., corazonin, EH, and α bursicon, were found in the transcriptome of adult, female *Ixodes scapularis* ticks [24], and corazonin, α and β bursicon, and CCAP messages were found in adult females of *Ornithodoros turicata* ticks [24]. Regardless of whether the full or partial presence of the molting pathway was found in different species of adult ticks, their detection in adults suggests they may have other functions besides molting.

In the current study, we examined the sequence homology and phylogeny of corazonin, eclosion hormone, and bursicon from adult *D. variabilis* female synganglia to that found in other ticks, larval insects, and other arthropods; message expression levels during *D. variabilis* female reproduction; and whether suppression of the corazonin message by RNAi affects host-seeking, partial feeding, mating, feeding to repletion, separation from the host, yolk deposition into eggs, and oviposition.

## 2. Materials and Methods

### 2.1. Ticks

American dog ticks, *Dermacentor variabilis*, were reared as described before [25]. The ticks were originally collected near Richmond, Virginia, USA. Adults were fed by confining them in a plastic capsule attached to New Zealand white rabbits, *Oryctolagus cuniculus*. Larvae and nymphs were fed on Norway rats, *Rattus norvegicus*, for laboratory colony maintenance and production of adults as needed. Rearing conditions were 26 ± 1 °C with a relative humidity of 92 ± 6% and a 14:10 L:D cycle. The use of all animals in this research were in accordance with protocols and recommendations of the Old Dominion University Institutional Animal Care and Use Committee (Animal Welfare Assurance Number: A3172-01). The approved protocols (#10-018 and #10-032) are on file in the Office of Research, Old Dominion University, Norfolk, Virginia, USA.

### 2.2. Synganglion Collection and Sample Preparation

The following developmental stages of adult, female *D. variabilis* were studied: (i) virgin, unfed 0–24 h after entering the adult stage (non-host-seeking), (ii) host-seeking, unfed, and not mated (3 d after emergence), (iii) part-fed (unmated, attached to host; 1st and 3rd day after emergence), (iv) mated (females are part-fed; allowed to mate for ≤1 day, 7th day after emergence), (v) mated repletes (completion of blood feeding but still attached to host), and (vi) post-drop-off (from host) with egg laying starting within 1 d of detachment. The synganglia were dissected intact from the ticks’ bodies (a dorsal dissection) and collected in phosphate-buffered saline (PBS) (pH 7.0, 10 mM NaH_2_PO_4_, 150 mM NaCl) on ice. Five biological replicate experiments were conducted for each of the above stages, where each biological replicate was pooled from five synganglia from each stage. Pooled tick synganglia were homogenized in Qiagen RLT buffer, and total RNA was isolated using an RNAeasy mini kit in accordance with the manufacturer’s recommendations (Qiagen, Germantown, MD, USA). The purity and concentration of RNA were determined with a Nanodrop 2000 spectrophotometer (Thermo fisher, Wilmington, DE, USA). All the samples were stored at −80 °C for further analysis. cDNA was prepared with superscript II (Invitrogen, Thermo fisher Scientific, Waltham, MA, USA). cDNA synthesis in 15 µL was conducted with 200 ng of total RNA for each reaction. The cDNA synthesis program was run at 25 °C for 10 min, 42 °C for 50 min, and 70 °C for 10 min on a Bio-Rad thermocycler PTC-200 (BioRad Laboratories, Hercules, CA, USA).

### 2.3. Bioinformatics

The sequences for female *D. variabilis* synganglion corazonin, eclosion hormone, α bursicon, and β bursicon transcripts have been described before by our team [22,26]. Briefly, PCR was conducted on a Bio-Rad thermocycler PTC-200 (Bio-Rad Laboratories, Hercules, CA, USA) using GoTaq Green Master Mix (Promega, Madison, WI, USA) according to the manufacturer’s recommendations using the primers in Table 1. These same primers were used for qPCR, as described later. The PCR cycling conditions were 95 °C for 2 min followed by 25 cycles of 95 °C for 30 s, 65 °C for 1 min, and 72 °C for 2 min. Amplified products were Sanger-sequenced at Eton Biosciences, Inc. (Research Triangle Park, NC, USA).

The sequences for corazonin, eclosion hormone, α bursicon, and β bursicon are shown in Figures S1A, S2A, S3A and S3B, respectively. The alignments of these putative *D. variabilis* synganglion messages (Figures S1A, S2A, S3A and S3B, respectively) and their phylogenies (Figures S1B, S2B, S3C and S3D, respectively) were investigated using available sequence data in GenBank. BLASTx [27] searches were conducted against the NCBI database, and sequence alignments were performed with the multi-alignment ClustalW software package (https://www.genome.jp/tools-bin/clustalw; accessed on 3 July 2023) [28] with messages for tick, insect, and other-arthropod putative neurohormone messages with high similarity. We used different genera in some cases with lower percent identity and query coverage to understand genetic variability between taxa. Since the data available were limited, we could not conduct alignments with the same organisms for each of the putative *D. variabilis* corazonin, eclosion hormone, α bursicon, and β bursicon putative messages. Trees were constructed only in the area where contiguous sequence information was found in the alignments (where gaps were minimal). Phylogenetic trees were constructed by the neighbor-joining [29] method with MEGA11 [30]. The robustness of tree topologies was analyzed with Bootstrap analyses, consisting of 1000 iterations. The *D. variabilis* messages share high similarity to similar messages in GenBank with the same functional assignments and are discussed in Supplementary Materials. However, the absolute functional assignment in ticks is unknown.

### 2.4. Quantitative PCR (qPCR) and Analysis

Relative expression of corazonin, eclosion hormone, α bursicon, and β bursicon messages were accessed by qPCR at different developmental stages of adult, female *D. variabilis* (described earlier) to understand their possible role in adult, female development and reproduction. The expression of corazonin was also determined by qPCR in corazonin suppression experiments (described later). From each sample, 2 µL of cDNA was used for qPCR with SYBR green master mix (Applied Biosystem, Carlsbad, CA, USA). qPCR reactions were carried out on a Bio-Rad CFX348 thermocycler (Bio-Rad Laboratories, Hercules, CA, USA). Reactions were run with an initial start of 95 °C for 3 min, followed by 39 cycles of 95 °C for 3 s and 63 °C for 10 s. A melt curve was conducted at the end of the run. Each biological replicate was performed in triplicate. Gene expression was normalized to glyceraldehyde 3-phosphate dehydrogenase (GAPDH) and analyzed by the 2^−ΔΔCT^ method [31]. The primers used for qPCR are shown in Table 1.

### 2.5. RNA Interference (RNAi)

Since corazonin is the first message that initiates the peptidic hormonal cascade for ecdysis in insects [8], RNAi was conducted to determine if the suppression of this message would affect female tick reproduction. The forward (5′-TAATACGACTCACTATAGGGGAGACCAGAACTAGCAGACAAACG-3′) and reverse (5′-TAATACGACTCACTATAGGGCAGCAGGCGACCCTTACG-3′) PCR primers for corazonin were used to synthesize the sense and antisense strands of the cDNA with T7 promoter regions included in the PCR reaction. dsRNA was synthesized by using a high-yielding MEGAscript^®^ RNAi kit (Ambion Inc., Austin, TX, USA). dsRNA was also constructed for male engorgement factor alpha (efα) message with forward (5′-TAATACGACTCACTATAGGGCGCGATTCCCGTGTACGAT-3′) and reverse (5′-TAATACGACTCACTATAGGGGCGCCGGTTCCTCTGCAG-3′) PCR primers as a study control. Efα messages are present in adult males [5]. The dsRNA final dilution was in 50 µL of PBS (described earlier). Water, eclosion hormone, and PBS were also used as controls in the RNAi experiments.

Newly emerged (0–24 h old after emergence) adult *D. variabilis* (non-host-seeking) females were each injected with 2 µL of ~250 ng of dsRNA/μL, using a 50 μL Hamilton syringe fitted with a 1.25 cm, 30-gauge needle. The dsRNA was injected into the dorsal posterior region of the female body. The inserted needle was held in place for 10–15 s before withdrawal to minimize leakage. Controls were also injected the same way. After injection, the ticks were placed on the host (a New Zealand white rabbit) and allowed to mate (a 1.5:1.0 male–female ratio) and were fed to repletion. The ticks were monitored to ensure that mating occurred (by observing the transfer of a spermatophore). The results are for mated, engorged females only. Engorged ticks were transferred to an incubator under environmental conditions described earlier to observe oviposition and egg development (by a dorsal dissection exposing the ovaries).

This study was conducted in duplicate on different days. In the first experiment, the injections were with corazonin dsRNA, eclosion hormone dsRNA, or PBS along with untreated ticks. Forty ticks were injected for each treatment, and the ticks were observed for percent oviposition. In the second experiment, ninety ticks were each injected with corazonin dsRNA, Efα dsRNA, or water. Efα was used as another control, since this message and protein are only found in male ticks [5,32,33]. The ticks were observed for percent oviposition or dissected to determine the number of vitellogenic (brown) eggs in the ovaries (Roe et al. [5]). Vitellgenin incorporates heme, making the protein brown, and, when concentrated into eggs in the ovaries as vitellin, changes the eggs from white to brown [5].

### 2.6. Statistics

Statistical significance was determined by analysis of variance using the PROC generalized linear model (GLM) (SAS 9.3, SAS Institute, Inc., Cary, NC, USA, 2003) with differences between two means determined by a *t*-test (α = 0.05).

## 3. Results

### 3.1. Expression of Corazonin at Different Stages

**Figure 1:**
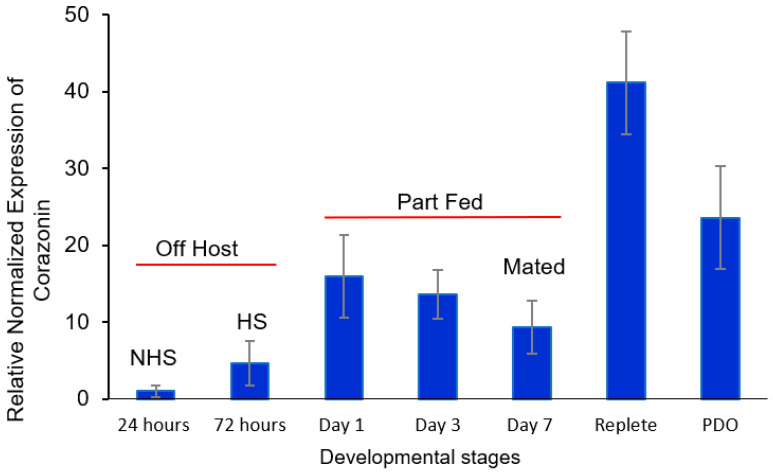
Relative expression of corazonin transcript levels at the following developmental stages of adult females of *Dermacentor variabilis*: (i) virgin, unfed 0–24 h after entering the adult stage (non-host-seeking), (ii) host-seeking, unfed, and not mated (3 d after emergence), (iii) part-fed (unmated, attached to host; 1st and 3rd day after emergence), (iv) mated (females are part-fed; allowed to mate for ≤1 day, 7th day after emergence), (v) mated repletes (completion of blood feeding but still attached to host), and (vi) post-drop-off (from host) with egg laying starting within 1 d of detachment. The error bars are ±1 standard error of the mean. HS, host-seeking; mated ≤1 day; NHS, non-host-seeking; PDO, post-drop-off.

The adult, female developmental stages of the American dog tick were described earlier (Section 2.2). Characterization of the adult *D. variabilis* transcripts for corazonin, eclosion hormone, and *α* and β bursicon compared to that in other tick species, insects, and other arthropods are described in Supplementary Materials (Figures S1–S3); they shared a high level of identity and reasonable phylogeny between the taxonomic groups that were examined. The message level for corazonin was the lowest just after entering the adult stage, with an increasing trend when they became host-seeking (not statistically significant, *t*-test, *α* = 0.05) (Figure 1). Transcript levels increased when first feeding till reaching the part-fed condition (day 1) compared to non-host-feeding ticks and then at an even higher level in mated, replete females compared to part-fed ticks (*t*-test, *α* = 0.05) (Figure 1). The expression of corazonin at this peak was 40-fold greater than at 24 h after emergence. The increases associated with partial feeding and especially associated with feeding to repletion suggest a function for the putative corazonin message in female development and during the time when the cuticle surface area of the tick is greatly expanding from the consumption of host blood. The trend was for the transcript levels to decrease after repletion when the ticks dropped from the host (Figure 1).

### 3.2. Expression of Eclosion Hormone at Different Developmental Stages

**Figure 2:**
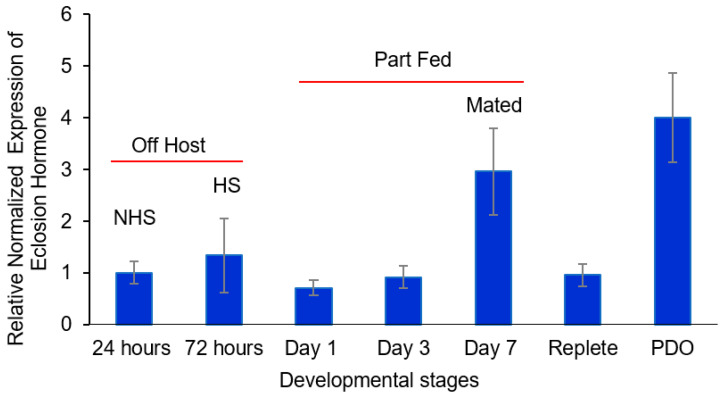
Relative expression of eclosion hormone at the following different developmental stages of adult, female *Dermacentor variabilis*: (i) virgin, unfed 0–24 h after entering the adult stage (non-host-seeking), (ii) host-seeking, unfed, and not mated (3 d after emergence), (iii) part-fed (unmated, attached to host; 1st and 3rd day after emergence), (iv) mated (females are part-fed; allowed to mate for ≤1 day, 7th day after emergence), (v) mated repletes (completion of blood feeding but still attached to host), and (vi) post-drop-off (from host) with egg laying starting within 1 d of detachment. The error bars are ±1 standard error of the mean. HS, host-seeking; mated ≤1 day; NHS, non-host-seeking; PDO, post-drop-off.

There was no significant difference in the message levels for the putative tick eclosion hormone transcript between just after emergence and when they became host-seeking at 72 h (Figure 2). There was a trend for an increase in levels between days 1 and 7, when the ticks acquired a host, part-fed, and then mated just prior to the big sip that was statistically significant during mating (*t*-test, *α* = 0.05). The levels decreased in replete ticks and then significantly increased (*t*-test, *α* = 0.05) after drop-off in anticipation of oviposition (Figure 2). Increased message levels were similar to corazonin (Figure 1) in that increases were associated with the period when mating, feeding to repletion, drop-off, and oviposition occur.

### 3.3. Expression of α and β Bursicon

**Figure 3:**
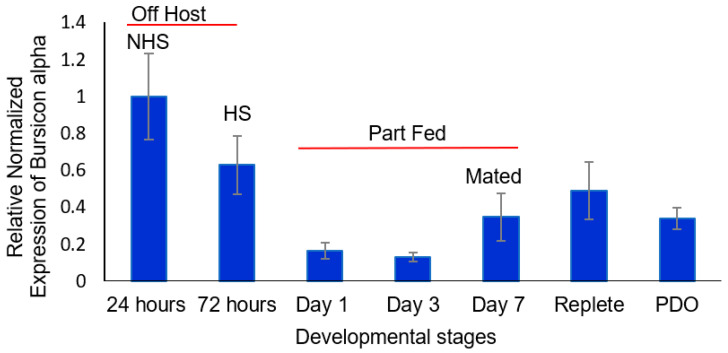
Relative expression of α bursicon at the following different developmental stages of adult, female *Dermacentor variabilis*: (i) virgin, unfed 0–24 h after entering the adult stage (non-host-seeking), (ii) host-seeking, unfed, and not mated (3 d after emergence), (iii) part-fed (unmated, attached to host; 1st and 3rd day after emergence), (iv) mated (females are part-fed; allowed to mate for ≤1 day, 7th day after emergence), (v) mated repletes (completion of blood feeding but still attached to host), and (vi) post-drop-off (from host) with egg laying starting within 1 d of detachment. The error bars are ±1 standard error of the mean. HS, host-seeking; mated ≤1 day; NHS, non-host-seeking; PDO, post-drop-off.

There was a higher level of *α* bursicon transcript 24 and 72 h after entering the adult stage compared to part-fed (unmated) females attached to the host (*t*-test, *α* = 0.05; Figure 3). The trend was a decreased level of transcript from 24 to 72 h for the unfed (virgin) females (not attached to the host) that continued after transfer to the host and partial feeding. Mating and blood feeding to repletion significantly increased *α* bursicon transcript levels compared to part-fed virgins, with a trend of a decrease after drop-off (not statistically significant) but with levels still higher than those for part-fed (virgin) ticks (days 1 and 3) (Figure 3).

**Figure 4:**
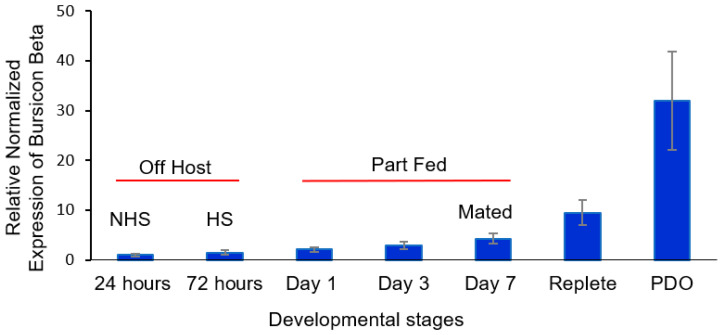
Relative expression of β bursicon at the following different developmental stages of adult females of *Dermacentor variabilis*: (i) virgin, unfed 0–24 h after entering the adult stage (non-host-seeking), (ii) host-seeking, unfed, and not mated (3 d after emergence), (iii) part-fed (unmated, attached to host; 1st and 3rd day after emergence), (iv) mated (females are part-fed; allowed to mate for ≤ 1 day, 7th day after emergence), (v) mated repletes (completion of blood feeding but still attached to host), and (vi) post-drop-off (from host) with egg laying starting within 1 d of detachment. The error bars are ±1 standard error of the mean. HS, host-seeking; mated 1 day; NHS, non-host-seeking; PDO, post-drop-off.

For β bursicon (Figure 4), there was a consistent trend of an increase in transcript levels from 24 h after molting to drop-off, but the changes were not statistically significantly different until after mating occurred on day 7. The transcript levels were statistically significantly higher (*t*-test, *α* = 0.05) for mated part-feds (day 7 ticks) compared to 24 h newly molted unfed ticks and were 32-fold higher after tick drop-off for ovipositing females. The increased levels of *α* and β bursicon (Figure 3 and Figure 4, respectively) after mating were similar to those of corazonin (Figure 1) and eclosion hormone (Figure 2), although the highest levels for all the transcript levels measured during this developmental period varied among mating, feeding to repletion, and drop-off.

### 3.4. Corazonin Message Suppression by RNAi and Effect on Egg Development

**Figure 5:**
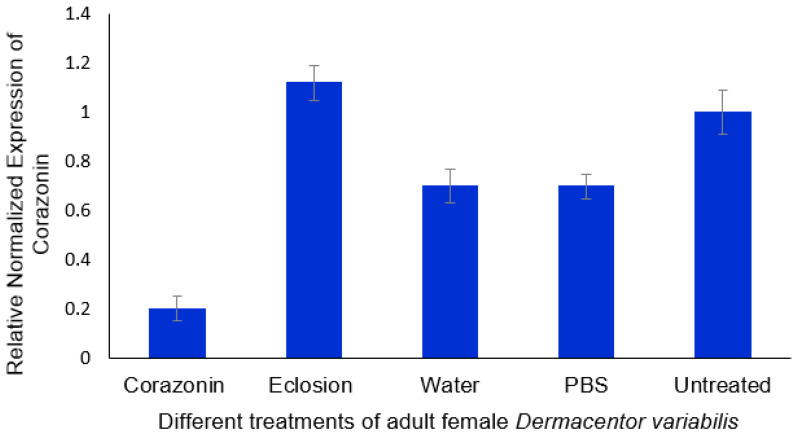
Relative expression of corazonin in dsRNA corazonin-treated adult *Dermacentor variabilis* as compared to controls. The error bars are ±1 standard error of the mean. PBS, phosphate-buffered saline.

Newly emerged (0–24 h old after emergence) adult *D. variabilis* (non-host-seeking) females were injected with corazonin dsRNA and compared to controls injected with eclosion hormone dsRNA, PBS, and water and to untreated ticks. The ticks were placed on a rabbit host after injection and allowed to mate and feed to repletion. The ticks were monitored to ensure that mating occurred. Corazonin transcript levels were then measured for the treatment and controls. The results are for mated females after feeding to repletion and release from the host. There was a clear reduction in the corazonin message levels compared to the other treatments (*t*-test, *α* = 0.05; Figure 5), with about a 5-fold decrease in corazonin as compared to the eclosion dsRNA-treated and untreated ticks. There was no difference between the eclosion hormone dsRNA-treated and untreated ticks. Corazonin message levels after water and PBS injections were less than those of the untreated control but significantly higher than the treatment (*t*-test, *α* = 0.05).

**Figure 6:**
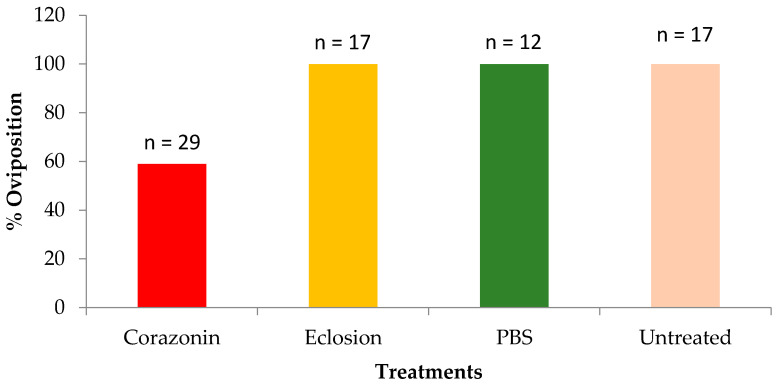
Percentage oviposition in adult, female *Dermacentor variabilis* treated with corazonin dsRNA, eclosion hormone dsRNA, or PBS (phosphate-buffered saline) and untreated adult, female ticks.

**Figure 7:**
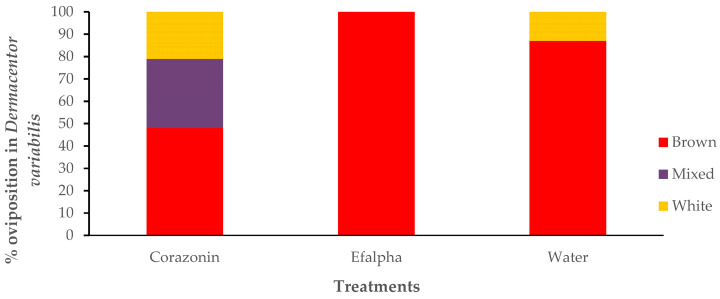
Percentage of *Dermacentor variabilis* ovaries with white, mixed (white and brown), and brown eggs after injection with corazonin dsRNA, Ef*α* dsRNA, or water.

Percentage oviposition was measured to investigate a possible biological effect of the corazonin suppression. All the control treatments demonstrated 100% oviposition, whereas the dsRNA corazonin-treated ticks showed 60% oviposition (Figure 6). To investigate the impact of dsRNA corazonin suppression on egg maturation, ticks were injected with dsRNA for corazonin, dsRNA for EFα, and distilled, sterile water. The ticks were placed on a rabbit host and allowed to mate and feed to repletion. The ovaries were dissected before oviposition. In the dsRNA corazonin treatment (n = 19), only 50% of the ovaries had brown, fully developed eggs, and 28% had a mixture of white and brown eggs (Figure 7). In the Ef*α* treatment (n = 6), all of the eggs were brown, and 85% were brown in the water control (n = 15) (Figure 7).

## 4. Discussion

After molting into the adult stage, American dog tick females are not host-seeking for the first day [34]. Afterward, they transit to finding a host. In our research, the host was a rabbit, and the ticks were placed on the rabbit. Once females are on the host, they attach, feed till reaching the part-fed condition, and then release pheromones to attract males already on the same host. During this period, the body volume increases about 10-fold but with no new cuticle synthesis occurring [35]. Mating for *D. variabilis* occurs only on the host. Males, after attaching, feed to completion, and then, in response to female pheromones [34], detach, locate the female, insert their mouthparts into the female genital tract, develop a spermatophore, and transfer the spermatophore to the female genital tract. The transfer of the spermatophore signals the female to feed to repletion, sometimes referred to as the “big sip”. Tick size increases dramatically, by a 100-fold, to accommodate the blood consumed [1]. Because the unfed, adult tick cuticle is folded, body expansion during partial feeding and feeding to repletion does not require the synthesis of new cuticle [35,36].

The spermatophore’s transfer into the female genital tract initiates, in the female, the synthesis of the steroid hormone 20-hydroxyecdysone (20H-E) [5,32,33]. This hormone initiates the synthesis and secretion of vitellogenin (a yolk protein) from the female fat body and midgut into the hemolymph and the synthesis of yolk protein receptors in the ovaries [5]. 20H-E does not initiate feeding to repletion. Vitellogenin in the hemolymph moves into eggs by receptor-mediated pinocytosis, after which the eggs are ready for fertilization and oviposition. The tick detaches from the host before releasing eggs. Ticks have lost the ability to synthesize heme [5]. Instead, they digest host blood-releasing heme, which is stored in their hemolymph. Vitellogenin in the hemolymph binds this heme, which is brown in color. When the yolk protein is incorporated into the eggs, they change from white to brown.

Other than the role of 20H-E in vitellogenesis, we know very little about the hormonal regulation of female development in ticks. The discovery of the transcript for corazonin from the synganglia of adult, female *D. variabilis* during the reproductive process [32] at the time was unexpected. One well-known function of the release of corazonin (initiated by decreasing 20H-E titers) in insects is to initiate a cascade of biochemical and behavioral changes that result in ecdysis, i.e., shedding of the old cuticle, followed by hardening (sclerotization) and darkening of the new cuticle [8]. Because adult ticks do not molt, the discovery of the corazonin message with a high similarity to the corazonin messages from several larval insects that molt (Figure S1) was unexpected. The phylogenetic analysis also showed that the *D. variabilis* corazonin message was most closely related to the cattle fever tick, *Rhipicephalus microplus*, and distantly related to mosquitoes and flies, as would be expected (with the exception of the moth *Bombyx mori* (Figure S1)).

Corazonin hormone is an undecapeptide originally described as a cardioaccelerator in the cockroach *Periplaneta americana* [10]. This peptide and its function in ticks is unknown. Similar peptides to that in the cockroach have been found in other insects: *Gryllus bimaculatus, Manduca sexta, Schistocerca americana* [11], *Bombyx mori* [37], and *Drosophila melanogaster* [38]. In the locust, it regulates the insect’s dark color, and in *B. mori,* it reduces silk spinning [12]. In male *D. melanogaster*, corazonin suppression blocks the transfer of sperm and seminal fluid and increases the duration of copulation [39]. In the ant *Harpegnathos saltator*, corazonin regulates caste differentiation and suppresses vitellogenesis [13]. Corazonin transcript levels in *D. variabilis* females increased from the point 24 h after ecdysis to the adult stage (non-host-seeking) to the start of partial feeding, and there was a second increase between day 7 (mated part-feds) and feeding to the replete condition, where the second period of increase was more dramatic (Figure 1). These developmental changes associated with feeding suggest that it has some association/role in adult, female development/feeding and likely has an impact on reproduction, since the nutrients from feeding are used to make yolk protein for egg development. The suppression of the corazonin message by RNAi reduced the percentage egg laying (Figure 6) and the number of brown (vitellogenic) eggs in the ovaries (Figure 7), further supporting this hypothesis.

This is the first evidence of a regulatory role of the tick corazonin message in female reproduction other than the message just being present in adults. It is not clear if the suppression reduced the amount of blood feeding, and this affected egg maturation. However, no differences in the size of the part-fed and replete ticks were apparent, suggesting that the transcript might have a function unrelated to regulating blood feeding specifically but is regulating the utilization of host blood, egg development, drop-off, and/or oviposition. This question of affecting the amount of feeding needs more research, however. Corazonin message suppression had no apparent effect on host attachment, part-feeding, their ability to attract a male and receive a spermatophore from the male into their genital track, blood feeding to repletion after mating, and detaching from the host. More research is needed to understand if ticks make the corazonin neuropeptide itself, its exact function in reproduction and whether corazonin functions with eclosion hormone, bursicon, and other neuropeptides (found in adult ticks) that control ecdysis in insects. The corazonin message was found in the *D. variabilis* synganglion, but the exact site of synthesis is unknown, and the hypothesis is that the synganglion is the only source for this transcript.

The presence of other messages in *D. variabilis* females associated with the regulation of insect ecdysis, e.g., eclosion hormone and α and β bursicon and their changes in message levels during adult development, also presents evidence that they have a role in tick reproduction (Figure 2, Figure 3 and Figure 4). These transcripts, like those for corazonin (Figure S1) discussed earlier, had a high similarity and expected phylogeny compared to transcripts from other ticks, insects, and other arthropods, as shown in Figures S2 and S3 and discussed in the Supplementary Materials related to these figures. Like corazonin, increased transcript levels for eclosion hormone were associated with the time of mating and feeding to repletion; the trend was that the highest levels were associated with detachment from the host (Figure 2). In insect molting, corazonin initiates a neuropeptide cascade that includes the release of eclosion hormone. Eclosion hormone initiates ecdysis behavior, i.e., the insect movement needed to escape from the old cuticle. One hypothesis might be that in ticks, eclosion hormone is involved in the 100-fold expansion of the tick body volume during feeding to repletion, the process for detachment from the host, and/or the behavior of oviposition. Tang et al. [40] suggested that an eclosion hormone-like gene might control female reproduction in the beetle *Tribolium castaneum*, having a role in tanning.

The developmental changes in α bursicon versus β bursicon early in adult development were different, the former dramatically decreasing in transcript levels (Figure 3) and the trend for β bursicon being a gradual increase (Figure 4). The α bursicon transcript levels peaked again after full engorgement, and β bursicon, which gradually increased throughout development, was highest at detachment from the host. Bursicon regulates sclerotization (cuticle hardening), darkening of the cuticle, and wing expansion after ecdysis [8,19], resulting from the same cascade that is initiated by corazonin and that initiates the earlier production of eclosion hormone in insects. Buriscon is important in the final stages of insect ecdysis in finishing the development of the new cuticle after ecdysis. However, in *D. melanogaster*, this hormone initiates an immune response against infection and stress by activating the NF-kB transcript factor [41]. Similarly, buriscon activates antimicrobial peptide genes in the cherry shrimp, *Neocaridina heteropoda* [21]. Tsutsui et al. [42] measured the expression of these neuropeptides in the thoracic ganglia and ovary transcriptomes of another shrimp species, *Marsupenaeus japonicus* [42], and suggested a possible role of bursicon in vitellogenin expression in the giant tiger prawn, *Penaeus monodon* (Sathapondecha et al. [43]). In *D. melanogaster*, β bursicon alone is a signal for controlling ecdysis behavior, whereas α bursicon signals the preservation of energetic homeostasis independent of β bursicon [44].

Our research shows that α and β bursicon levels can change independently of each other (compare Figure 3 with Figure 4), suggesting possible different functions for these messages, although they are also known in insects to be present as a heterodimer [19,20,21]. Our hypothesis is that bursicon has some role in egg and/or eggshell formation, since, in insects, bursicon regulates cuticle maturation; adult *D. variabilis* females at this time are not developing new cuticle but are developing and releasing eggs. There is some evidence from our study that at least α bursicon may have a role in cuticle maturation in ticks after ecdysis to the adult stage, since the transcript levels were highest just after molting from the nymph to the adult (Figure 3).

The roles of corazonin, eclosion hormone, and bursicon have never been studied relative to tick molting [1]. Based on the developmental expression data in this current study, it appears that eclosion hormone and bursicon have no role in attachment and partial feeding, since no significant increases in transcript levels were found.

## 5. Conclusions

Increases in putative corazonin, eclosion hormone, α bursicon, and β bursicon are associated with blood feeding, especially after mating and the initiation of feeding to repletion in adult, female American dog ticks. The transcripts had a high level of similarity to those in insects and other arthropods. Our phylogenetic analysis also showed that each hormone in *D. variabilis* was most closely related to that in the Acari compared to other arthropods. This is the first time that the level of these transcripts has been examined during adult tick development and reproduction. Although these transcripts are involved in the regulation of ecdysis in insects, they must have a different role in adult ticks since adults do not molt. Corazonin initiates the cascade that leads to ecdysis in insects; however, in ticks, it leads to reduced oviposition success and the number of brown, vitellogenic eggs in the ovaries. Changing levels of eclosion hormone were hypothesized to have a function in feeding to repletion, detachment from the host, and/or oviposition and, for bursicon, in the maturation of the eggshell. However, much more research is needed to understand the roles of these “homologous” molecules in tick endocrinology, reproduction, and oviposition, where the functions are distinctly different from their roles in insects to regulate molting and juvenile development.

## Figures and Tables

**Table 1:** Primers used for PCR and qPCR of *D. variabilis* corazonin, α bursicon, β bursicon, and eclosion hormone.

Gene of Interest	Primers	Sequence	Product Size (Base Pairs)
Corazonin	Forward	GAGACCAGAACTAGCAGACAAACG	59
	Reverse	CAGCAGGCGACCCTTACG	
α Bursicon	Forward	GCATGTGCTGCCAGGAGAT	90
	Reverse	TGACCAGCTTGCGGAACTT	
β Bursicon	Forward	ACGAAATTTCCAAATCGCATCT	80
	Reverse	GTTGAGTCTGCCCCAGTATCG	
Eclosion Hormone	Forward	TGCGCCGAGGAGTGTGT	54
	Reverse	GATGTCGCAGTCAGGTTGGA	

## Data Availability

Data are available upon request with justification from the corresponding author.

## Supplementary Materials

The following supporting information can be downloaded at https://www.mdpi.com/article/10.3390/insects16040343/s1, References [45,46] are cited in Supplementary Materials.

## References

[1] Sonenshine D.E., Roe R.M. (2014). Overview: Ticks, people, and animals. Biology of Ticks.

[2] Lees K., Bowman A.S. (2007). Tick neurobiology: Recent advances and the post-genomic era. Invertebr. Neurosci..

[3] Guerrero F., Pérez de León A., Rodriguez-Vivas R., Jonsson N., Miller R., Andreotti R., Sonenshine D., Roe R. (2014). Acaricide research and development, resistance and resistance monitoring. Biology of Ticks.

[4] George J.E., Pound J.M., Davey R.B. (2004). Chemical control of ticks on cattle and the resistance of these parasites to acaricides. Parasitology.

[5] Roe R.M., Donahue K., Khalil S., Bissinger B.W., Zhu J., Sonenshine D.E. (2014). Hormonal regulation of metamorphosis and reproduction in ticks. Biology of Ticks.

[6] Ogihara M.H., Taylor D. (2014). Female reproductive system. Biology of Ticks.

[7] Neese P.A., Sonenshine D.E., Kallapur V.L., Apperson C.S., Roe R.M. (2000). Absence of insect juvenile hormones in the American dog tick, *Dermacentor variabilis* (Say) (Acari:Ixodidae), and in *Ornithodoros parkeri* Cooley (Acari:Argasidae). J. Insect Physiol..

[8] Klowden M.J., Palli S.R. (2023). Nervous Systems.

[9] Clark A.C., del Campo M.L., Ewer J. (2004). Neuroendocrine control of larval ecdysis behavior in Drosophila: Complex regulation by partially redundant neuropeptides. J. Neurosci..

[10] Veenstra J.A. (1989). Isolation and structure of corazonin, a cardioactive peptide from the American cockroach. FEBS Lett..

[11] Veenstra J.A. (1991). Presence of corazonin in three insect species, and isolation and identification of [His7]corazonin from *Schistocerca americana*. Peptides.

[12] Tanaka Y., Hua Y.J., Roller L., Tanaka S. (2002). Corazonin reduces the spinning rate in the silkworm, *Bombyx mori*. J. Insect Physiol..

[13] Gospocic J., Shields E.J., Glastad K.M., Lin Y., Penick C.A., Yan H., Mikheyev A.S., Linksvayer T.A., Garcia B.A., Berger S.L. (2017). The neuropeptide corazonin controls social behavior and caste identity in ants. Cell.

[14] Kim Y.-J., Spalovská-Valachová I., Cho K.-H., Zitnanova I., Park Y., Adams M.E., Zitnan D. (2004). Corazonin receptor signaling in ecdysis initiation. Proc. Natl. Acad. Sci. USA.

[15] Kataoka H., Troetschler R.G., Kramer S.J., Cesarin B.J., Schooley D.A. (1987). Isolation and primary structure of the eclosion hormone of the tobacco hornworm, *Manduca sexta*. Biochem. Biophy. Res. Commun..

[16] Kingan T.G., Gray W., Žitňan D., Adams M.E. (1997). Regulation of ecdysis-triggering hormone release by eclosion hormone. J. Exp. Biol..

[17] Žitňan D., Žitňanová I., Spalovská I., Takáć P., Park Y., Adams M.E. (2003). Conservation of ecdysis-triggering hormone signalling in insects. J. Exp. Biol..

[18] Roller L., Zitnanová I., Dai L., Simo L., Park Y., Satake H., Tanaka Y., Adams M.E., Zitnan D. (2010). Ecdysis triggering hormone signaling in arthropods. Peptides.

[19] Song Q. (2012). Bursicon, a neuropeptide hormone that controls cuticletanning and wing expansion. Insect Endocrinology.

[20] Srivastava B.B.L., Hopkins T.L. (1975). Bursicon release and activity in haemolymph during metamorphosis of the cockroach, *Leucophaea maderae*. J. Insect Physiol..

[21] Li R., Weng J., Wang X., Meng Q., Wang Y., Sun J. (2019). Bursicon homodimers induce innate immune by activating the expression of anti-microbial peptide genes in the shrimp *Neocaridina heteropoda*. Fish Shellfish Immunol..

[22] Donohue K.V., Khalil S.M.S., Ross E., Grozinger C.M., Sonenshine D.E., Michael Roe R. (2010). Neuropeptide signaling sequences identified by pyrosequencing of the American dog tick synganglion transcriptome during blood feeding and reproduction. Insect Biochem. Mol. Biol..

[23] Waldman J., Xavier M.A., Vieira L.R., Logullo R., Braz G.R.C., Tirloni L., Ribeiro J.M.C., Veenstra J.A., da Silva Vaz I. (2022). Neuropeptides in *Rhipicephalus microplus* and other hard ticks. Ticks Tick-Borne Dis..

[24] Egekwu N., Sonenshine D., Garman H., Barshis D., Cox N., Bissinger B., Zhu J., Roe R.M. (2016). Comparison of synganglion neuropeptides, neuropeptide receptors and neurotransmitter receptors and their gene expression in response to feeding in *Ixodes scapularis* (Ixodidae) vs. *Ornithodoros turicata* (Argasidae). Insect Mol. Biol..

[25] Daniel S. (1993). Biology of Ticks.

[26] Bissinger B.W., Donohue K.V., Khalil S.M.S., Grozinger C.M., Sonenshine D.E., Zhu J., Roe R.M. (2011). Synganglion transcriptome and developmental global gene expression in adult females of the American dog tick, *Dermacentor variabilis* (Acari: Ixodidae). Insect Mol. Biol..

[27] Altschul S.F., Madden T.L., Schäffer A.A., Zhang J., Zhang Z., Miller W., Lipman D.J. (1997). Gapped BLAST and PSI-BLAST: A new generation of protein database search programs. Nucleic Acids Res..

[28] Larkin M.A., Blackshields G., Brown N.P., Chenna R., McGettigan P.A., McWilliam H., Valentin F., Wallace I.M., Wilm A., Lopez R. (2007). Clustal W and Clustal X version 2.0. Bioinformatics.

[29] Saitou N., Nei M. (1987). The neighbor-joining method: A new method for reconstructing phylogenetic trees. Mol. Biol. Evol..

[30] Tamura K., Stecher G., Kumar S. (2021). MEGA11: Molecular evolutionary genetics analysis version 11. Mol. Biol. Evol..

[31] Livak K.J., Schmittgen T.D. (2001). Analysis of relative gene expression data using real-time quantitative PCR and the 2^−ΔΔCT^ method. Methods.

[32] Donohue K.V., Khalil S.M., Ross E., Mitchell R.D., Roe R.M., Sonenshine D.E. (2009). Male engorgement factor: Role in stimulating engorgement to repletion in the ixodid tick, *Dermacentor variabilis*. J. Insect Physiol..

[33] Sonenshine D.E., Bissinger B.W., Egekwu N., Donohue K.V., Khalil S.M., Roe R.M. (2011). First transcriptome of the testis-vas deferens-male accessory gland and proteome of the spermatophore from *Dermacentor variabilis* (Acari: Ixodidae). PLoS ONE.

[34] Sonenshine D.E., Silverstein R.M., West J.R. (1984). Occurrence of sex attractant pheromone, 2,6-dichlorophenol, in relation to age and feeding in American dog tick, *Dermacentor variabilis* (say) (Acari:Ixodidae). J. Chem. Ecol..

[35] Lees A.D. (1952). The role of cuticle growth in the feeding process of ticks. Proc. Zool. Soc. Lond..

[36] Hackman R.H., Filshie B.K. (1982). The Tick Cuticle. Physiology of Ticks.

[37] Hua Y.-J., Ishibashi J., Saito H., Tawfik A.I., Sakakibara M., Tanaka Y., Derua R., Waelkens E., Baggerman G., De Loof A. (2000). Identification of [Arg7] corazonin in the silkworm, *Bombyx mori* and the cricket, *Gryllus bimaculatus*, as a factor inducing dark color in an albino strain of the locust, *Locusta migratoria*. J. Insect Physiol..

[38] Veenstra J.A. (1994). Isolation and structure of the *Drosophila* corazonin gene. Biochem. Biophys. Res. Commun..

[39] Tayler T.D., Pacheco D.A., Hergarden A.C., Murthy M., Anderson D.J. (2012). A neuropeptide circuit that coordinates sperm transfer and copulation duration in *Drosophila*. Proc. Natl. Acad. Sci. USA.

[40] Tang J., Yu R., Zhang Y., Xie J., Song X., Feng F., Gao H., Li B. (2022). Molecular and functional analysis of eclosion hormone-like gene involved in post-eclosion behavior in a beetle. J. Insect Physiol..

[41] An S., Dong S., Wang Q., Li S., Gilbert L.I., Stanley D., Song Q. (2012). Insect neuropeptide bursicon homodimers induce innate immune and stress genes during molting by activating the NF-κB transcription factor Relish. PLoS ONE.

[42] Tsutsui N., Kobayashi Y., Izumikawa K., Sakamoto T. (2020). Transcriptomic analysis of the kuruma prawn *Marsupenaeus japonicus* reveals possible peripheral regulation of the ovary. Front. Endocrinol..

[43] Sathapondecha P., Panyim S., Udomkit A. (2015). A novel function of bursicon in stimulation of vitellogenin expression in black tiger shrimp, *Penaeus monodon*. Aquaculture.

[44] Zhou Y., Nagata S. (2021). Bursicon.

[45] Truman J.W., Taghert P.H., Copenhaver P.F., Tublitz N.J., Schwartz L.M. (1981). Eclosion hormone may control all ecdyses in insects. Nature.

[46] Davis M.M., O’Keefe S.L., Primrose D.A., Hodgetts R.B. (2007). A neuropeptide hormone cascade controls the precise onset of post-eclosion cuticular tanning in Drosophila melanogaster. Development.

