# Supplementary material for: Developmental Regulation of Corazonin, Eclosion Hormone, and Bursicon Messages and RNAi Suppression of Corazonin in Adult, Female American Dog Ticks, Dermacentor variabilis
Source: Insects. 2025 Mar 25;16(4):343. doi: 10.3390/insects16040343 (PMC12027769; doi:10.3390/insects16040343)
Supplement: Supplementary file 1 [file insects-16-00343-s001.zip › insects-3461448-supplementary.pdf]

Multiple sequence alignments of molting pathway hormones

Corazonin: A highly conserved corazonin neuropeptide message was identified by our laboratory from the synganglion of female adults of *D. variabilis* [1], a 545 bp transcript with a unique signal peptide. In our analysis here, Corazonin from *D. variabilis* was aligned at the amino acid level with multiple corazonin transcripts from another tick species and with different insect orders. Multiple sequence alignments and phylogenetics (Figure S1) were conducted to further support the putative identity assignment of corazonin in the American dog tick. Studies of the corazonin transcript are most advanced in the Insecta. Corazonin from *D. variabilis* (Class Arachnida, Order Ixodida, Family Ixodidae) was aligned with that from *Aedes aegypti* (Class Insecta, Order Diptera, Family Culicidae), *Bombyx mori* (Class Insecta, Order Lepidoptera, Family Bombycidae), *Drosophila melanogaster* (Class Insecta, Order Diptera, Family Drosophilidae) and *Rhipicephalus microplus* (Class Arachnida, Order Ixodida, Family Ixodidae). A conserve domain of identical amino acids was found in all insects and ticks (see box in Figure S1a). Corazonin from *D. variabilis* had its highest overall sequence homology with *Rhipicephalus microplus* with an E-value of 7e-48 and percent identity of 80.68%. The phylogenetic tree (Figure S1b) shows the relatedness of the transcripts. Corazonin as expected showed greater divergence between ticks and insects. The divergence between ticks and *B. mori* was less than expected, since they were between different classes and greater than expected between *B. mori* and the other insects considering that all of the insects were in the Holometabola. However, absolute identity between ticks and insects in the conserved region along with the phylogeny argue that the assignment of the *D. variabilis* transcript as corazonin was reasonable. Corazonin is a undecapeptide hormone which was originally discovered as a cardioaccelerator hormone in the cockroach, *Periplaneta americana* (Class Insecta, Order Blattodea, Family Blattidae) [2]. Declining levels of 20-ecdysone after apolysis is completed triggers the release of corazonin from Inka cells which in turn releases ecdysis-triggering hormone from the Inka cells followed by the release of eclosion hormone from the insect brain [3].

```
Aedes            MK-HVFSTSLIVSLFVIFTDAQTFQYSRGWTNGKRSSPEQTAP-SRTLLPHIPLGMDKPD
Bombyx           MVTNI--TLILTLMTLASVTAQTFQYSRGWTNGKRDGHKRDELRDEV------LERI--L
Drosophila       MLRLLL--LPLFLFTLSMCMGQTFQYSRGWTNGKRSFNAASPLLANGHL-HRASELG--L
Dermacentor      MSRTVATCGVLLACLVMIASCQTFQYSRGWTNGKRRDGAIVAGPSRVTVEHRLLEEF--L
Rhipicephalus    MSRIVATFGLLLFNLVMIVHCQTFQYSRGWTNGKRRDGPIVAVPSRITADHRLLEEF--L
                 *   :     :    :     **************

Aedes            EECRLLIQRFLKSPCDVRLANAIVNRNKDLLRDMADDVN---------------------
Bombyx           TPCQLDKLKYV--LEG----KPLN---------------DRLFVP-CDYIEEEVN-----
Drosophila       TD------LYD--LQD----WSSDRRLERCLSQLQRSLIARNCVPGSDFNANRVDPDPEN
Dermacentor      S-------KFA--PKD----RVVLERLGHLFRTLDRSEDDQE------Y-----------
Rhipicephalus    S-------KFA--PKD----RVIVERLGHLLRTLDRNEDEQE------Y-----------

Aedes            ---------DGTALLYDPVPMVDTAASED-----VRFKRGTP-DRRLLNDGMHRL   137
Bombyx           --QPKRYKGERNHELFDVFQ-----------------------------------   101
Drosophila       SAHPRLSNSNGENVLYSSANIPNRHRQSNELLEELSAAGGASAEPNVF--GKH--   154
Dermacentor      -------------------------------------------------------   88
Rhipicephalus    -------------------------------------------------------   88
```

Figure S1a. Multiple alignments at the transcript level for corazonin from *Dermancentor variabilis* against that for another tick species and insects. Boxed section was a conserved domain with 100% identity between ticks and insects. The accession numbers are shown in Figure S1b. Asterisks indicate absolute identify with highly similar residues.

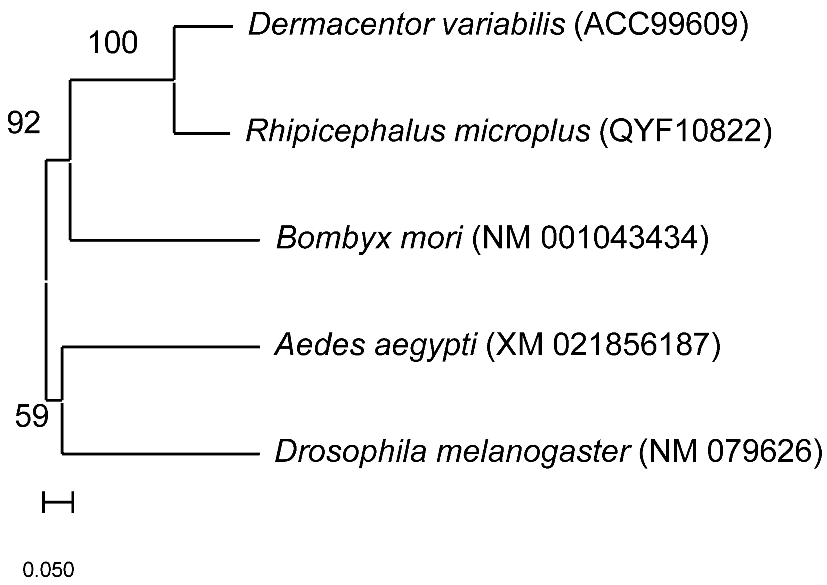

Figure S1b. Phylogenetic tree showing the evolutionary relationship between the *D. variabilis* corazonin transcript to that for another tick species and insects. The accession number for each is in parentheses. Numbers at the nodes indicate levels of bootstrap support (%). Only values above 50% are shown. Bar, 0.050 substitutions per nucleotide.

Eclosion Hormone: The eclosion hormone transcript was identified in *D. variabilis* by the presence of the conserved region from residues 12-64 (see box, Figure S2a). Eclosion hormone from *D. variabilis* was aligned with *Cheraz quandricarinatus* (Class Malacostraca, Order Decapoda, Family Parastacidae), *Blattella germanica* (Class Insecta, Order Blattodea, Family Ectobiidae), *Phtinus pyralis* (Class Insecta, Order Coleoptera, Family Lampyridae), *Aphis mellifera* (Class Insecta, Order Hymenoptera, Family Apidae), and *Rhipicephalus sanguineus* (Class Arachnida, Order Ixodida, Family Ixodidae). Most of the residues in this

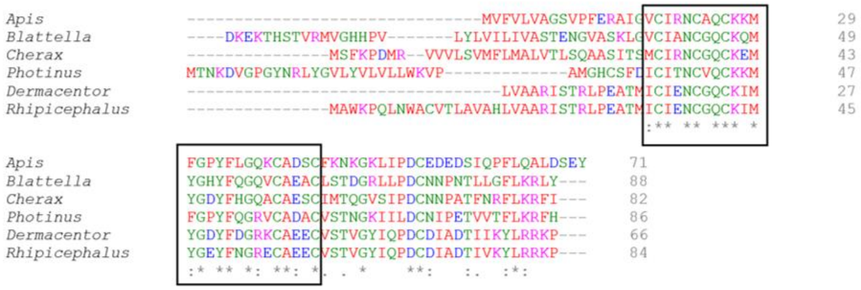

Figure S2a. Multiple alignments at the transcript level for eclosion hormone from *Dermacentor variabilis* against that for another tick species, insects and a decapod. Boxed section is a highly conserved region in the alignments. The accession numbers are shown in Figure S2b. Asterisks indicate absolute identify with highly similar residues donated by a colon.

motif were fully conserved, designated by asterisks with some of them with highly similar residues donated by a colon. This is the hormone that initiates ecdysis behavior in insects [4]. The eclosion hormone

transcript from *D. variabilis* best matched to that of *R. microplus* with an E-value of 6e-39 and a percent identity of 94.93%. However, even distant related insects like *Apis mellifera* (Figure S2a) had an E-value of 1e-08 and a percent identity of 43.86% unlike the results for corazonin where similar comparisons had higher E-values. Eclosion hormone in insects along with other hormones induces pre-ecdysis and ecdysis behavior [3,5] followed by the release of bursicon [6].

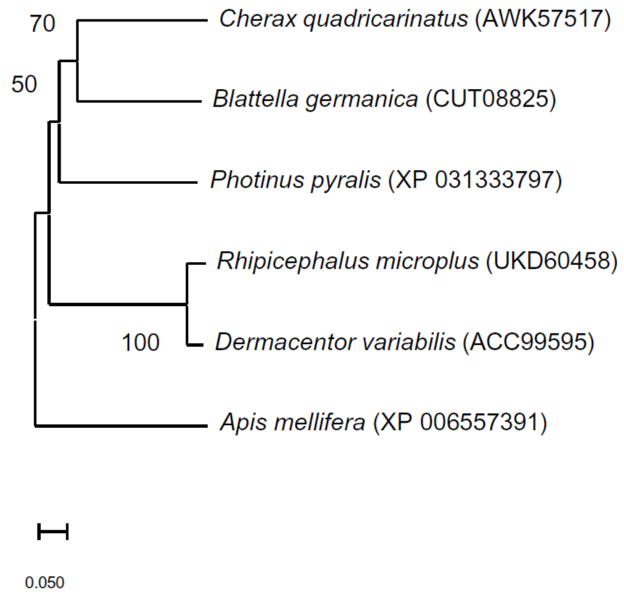

Figure S2b. Phylogenetic tree showing the evolutionary relationship between the *D. variabilis* eclosion hormone transcript to that for another tick species, insects and a decapod. The accession number for each is in parentheses. Numbers at the nodes indicate levels of bootstrap support (%), only values above 50% are shown. Bar, 0.050 substitutions per nucleotide.

Bursicon (α) and beta (β): α and β Bursicon were identified in adult *D. variabilis* as partial sequences. Bursicon α and β with 223 and 515 bp, respectively, were aligned in our study with *Caerostris darwini* (Class Arachnida, Order Araneae, Family Araneidae), *Daphania magna* (Class Branchiopoda, Order Anomopoda, Family Daphniidae), *Halyomorpha halys* (Class Insecta, Order Hemiptera, Family Pentatomidae), *Vespa mandarinia* (Class Insecta, Order Hymenoptera, Family Vespidae), *Cimex lectularius* (Class Insecta, Order Hemiptera, Family Cimicidae), *Locusta microtoria* (Class Insecta, Order Orthoptera, Family Acrididae), and *D. variabilis* and *Rhipicephalus sanguineus* (Class Arachnida, Order Ixodida, Family Ixodidae).

α Bursicon shows high conservation between different species (box in Figure S3a) with the top match with the tick, *R. microplus* (E-value 1e-47 and percent identity 80.00%). However, even distant arthropods like *Vespa mandarinia* (Figure S3a) had a low E-value (3e-23) and high percent identity (64.79%). As expected, the α bursicon transcripts from the chelicerates were grouped separately from that of mandibulates (Figure S3c).

β Bursicon was a longer sequence (515 bp) but with poor alignment with the other organisms (Figure S3b). The closely related tick, *R. microplus*, had an E-value of 6e-33 and percent identity of 95.24% but a low query coverage of 36%. The alignments shown in the most conserved region (box in Figure S3b) had a low level of conservation with absolute conversation shown with asterisks. Unexpected, the tick β bursicon transcript was grouped with the insect, *Locusta microtoria*, while the other mandibulates were grouped separate (Figure S3d).

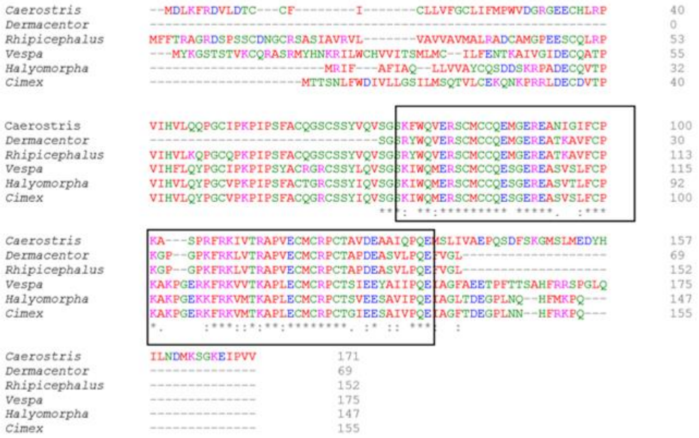

Figure S3a. Multiple alignments at the transcript level for α bursicon from *Dermacentor variabilis* against that for another tick species, insects and spiders. Boxed section is a highly conserved region in the alignments. The accession numbers are shown in Figure S3c. Asterisks indicate absolute identify with highly similar residues donated by a colon.

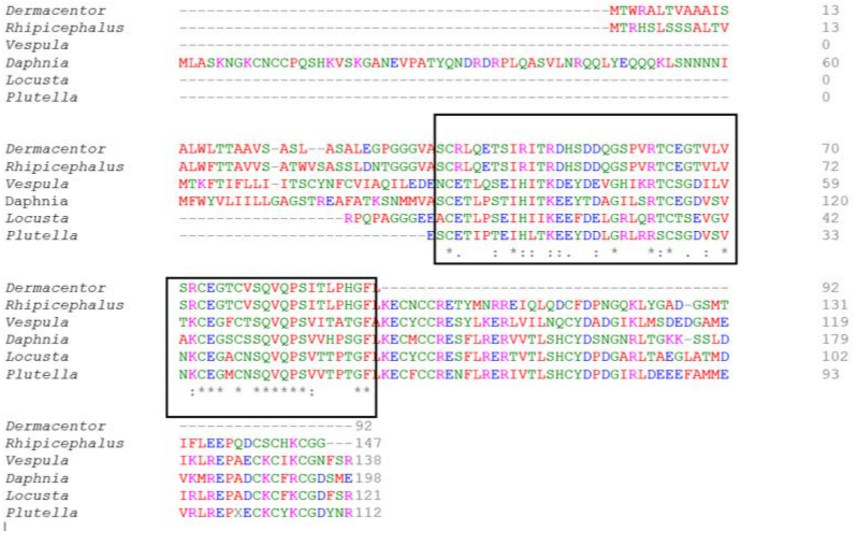

Figure S3b. Multiple alignments at the transcript level for β bursicon from *Dermacentor variabilis* against that for another tick species, insects and Branchiopoda. Boxed section is a highly conserved region in the alignments. The accession numbers are shown in Figure S3d. Asterisks indicate absolute identify with highly similar residues donated by a colon.

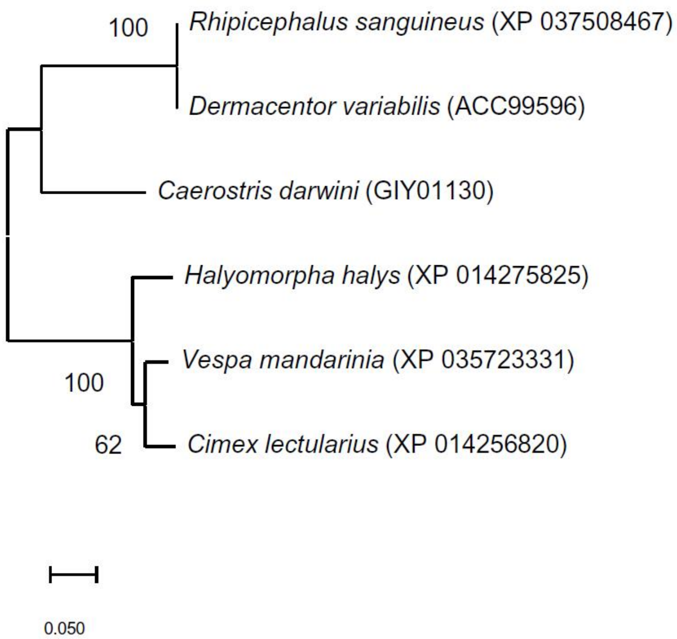

Figure S3c. Phylogenetic tree showing the evolutionary relationship between the *D. variabilis* α bursicon transcript to that for another tick species, insects and spiders. The accession number for each is in parentheses. Chelicerates (ticks and spiders) sorted separate from the insects. Numbers at the nodes indicate levels of bootstrap support (%), only values above 50% are shown. Bar, 0.050 substitutions per nucleotide.

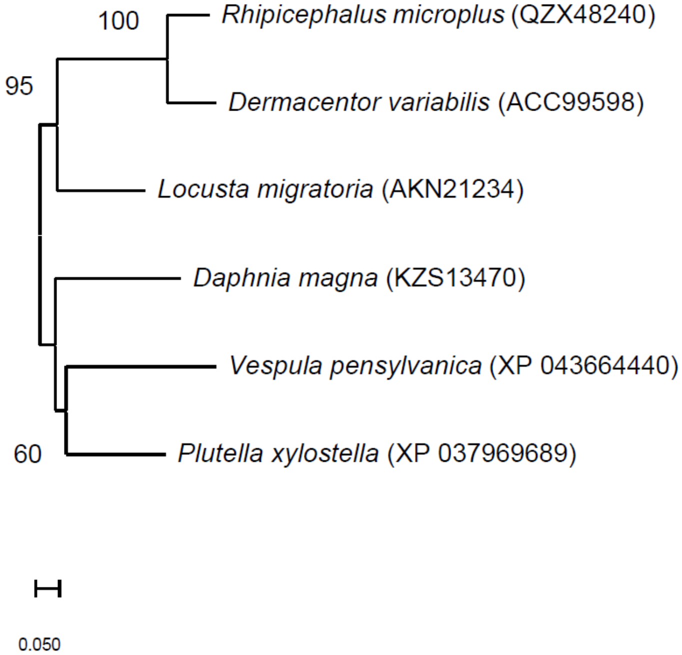

Figure S3d. Phylogenetic tree showing the evolutionary relationship between the *D. variabilis* β bursicon transcript to that for another tick species, insects and Branchiopda. The accession number for each is in parentheses. Numbers at the nodes indicate levels of bootstrap support (%), only values above 50% are shown. Bar, 0.050 substitutions per nucleotide.

In summary, the alignments and phylogenies for *D. variabilis* corazonin, eclosion hormone, α bursicon and β bursicon transcripts were most related to that from other tick species and mostly grouped separate compared to that from insects and other arthropods outside of insects and ticks, a reasonable outcome based on what we know about arthropod evolution. This grouping was also consistent with alignments for the tick corazonin, eclosion hormone, α bursicon and β bursicon transcripts with their respective counter parts in the better-studied insects. Corazonin, eclosion hormone, α bursicon and β bursicon that regulate insect molting demonstrated conservation between ticks, spiders, branchiopods, decapods and insects, which would be expected since ecdysis is a fundamental arthropod process associated with growth. However, the functional role of these putative tick transcripts, although associated as shown to the respective insect hormones that control molting, is unknown. These tick transcripts have not been studied in tick larvae and nymphs that molt. Their presence in female adult ticks that do not molt, suggest they may have an alternative role in blood feeding and/or reproduction and maybe in adult development in other arthropods.

References

1. Donohue, K.V.; Khalil, S.M.; Ross, E.; Mitchell, R.D.; Roe, R.M.; Sonenshine, D.E. Male engorgement factor: Role in stimulating engorgement to repletion in the ixodid tick, *Dermacentor variabilis*. *J. Insect Physiol.* **2009**, *55*, 909-918.
2. Veenstra, J.A. Isolation and structure of corazonin, a cardioactive peptide from the American cockroach. *FEBS Lett.* **1989**, *250*, 231-234, doi:10.1016/0014-5793(89)80727-6.
3. Klowden, M.J.; Palli, S.R. *Nervous systems*; Elsevier: 2023; pp. 527-605.
4. Truman, J.W.; Taghert, P.H.; Copenhaver, P.F.; Tublitz, N.J.; Schwartz, L.M. Eclosion hormone may control all ecdyses in insects. *Nature* **1981**, *291*, 70-71.
5. Davis, M.M.; O'Keefe, S.L.; Primrose, D.A.; Hodgetts, R.B. A neuropeptide hormone cascade controls the precise onset of post-eclosion cuticular tanning in *Drosophila melanogaster*. *Development* **2007**, *134*, 4395-4404.
6. Kim, Y.-J.; Spalovská-Valachová, I.; Cho, K.-H.; Zitnanova, I.; Park, Y.; Adams, M.E.; Zitnan, D. Corazonin receptor signaling in ecdysis initiation. *Proc. Natl. Acad. Sci. USA* **2004**, *101*, 6704-6709, doi:10.1073/pnas.0305291101.
